# Re-Thinking Pharmacokinetics in Ovarian Cancer: What Do Organoids Add?

**DOI:** 10.3390/ijms27083423

**Published:** 2026-04-10

**Authors:** Ana Emanuela Cisne de Lima, Mariana Nunes, Cristina P. R. Xavier, Sara Ricardo

**Affiliations:** 1 Applied Molecular Biosciences Unit (UCIBIO), Toxicologic Pathology Research Laboratory, University Institute of Health Sciences, Cooperative for Polytechnic and University Higher Education (1H-TOXRUN, IUCS-CESPU), 4585-116 Gandra, Portugal; cisneemanuela@gmail.com (A.E.C.d.L.); marianaoliveiranunes1992@gmail.com (M.N.); cristina.xavier@iucs.cespu.pt (C.P.R.X.); 2 Associate Laboratory of Institute for Health and Bioeconomy (i4HB), University Institute of Health Sciences, Cooperative for Polytechnic and University Higher Education (IUCS-CESPU), CRL, 4585-116 Gandra, Portugal; 3 Institute for Research and Innovation in Health (i3S), University of Porto, 4200-135 Porto, Portugal

**Keywords:** ovarian cancer, patient-derived organoids, pharmacokinetics, pharmacodynamics, PBPK, IVIVE, malignant ascites

## Abstract

Ovarian cancer (OC) remains one of the leading causes of gynecologic cancer mortality, largely due to late diagnosis, frequent relapse, and the emergence of chemoresistance. An important but often-overlooked contributor to treatment failure is the heterogeneous penetration of anticancer drugs within tumors. Structural and biochemical barriers—including abnormal vasculature, elevated interstitial pressure, dense extracellular matrix, drug efflux transporters, and malignant ascites—generate steep intratumoral concentration gradients that conventional preclinical models fail to capture. As a result, systemic pharmacokinetic measurements frequently provide limited insight into tumor-level drug exposure. Patient-derived organoids (PDOs) have emerged as physiologically relevant 3D models that preserve the genetic, architectural, and functional characteristics of the original tumor. These systems enable controlled investigation of pharmacokinetic and pharmacodynamic processes, including drug penetration, metabolism, retention, and exposure–response relationships. Adding cell-free malignant ascites supernatant enhances PDOs’ ability to mimic the metastatic peritoneal microenvironment of OC. This review discusses recent advances in PDO technologies and examines how PDO-derived data can inform intratumoral pharmacokinetics and dosing strategies using physiologically based pharmacokinetic modeling and *in vitro–in vivo* extrapolation. Emerging hybrid platforms, including organoid-on-chip systems, vascularized co-cultures, and multi-omics integration, are crucial to improve translational prediction and support precision oncology.

## 1. Introduction

Ovarian cancer (OC) has high mortality rates mainly due to late-stage diagnosis, high rates of recurrences, and emergence of chemoresistance [1,2]. In 2020, OC accounted for about 3.7% of newly diagnosed female cancers and 4.7% of cancer deaths worldwide [1]. Historically, the highest rates have been observed in Northern Europe and North America; however, changing classification practices—such as attributing many cases to fallopian tube origin—and shifting risk factor patterns have altered geographical incidence [2].

OC is a highly heterogeneous disease encompassing several histopathological and molecular subtypes, each defined by distinct genomic alterations and clinical trajectories. Among epithelial OC, high-grade serous carcinoma (HGSC) is the most common subtype [3,4]. Within the global burden, histotype-specific biology—dominated by HGSC—shapes the therapeutic challenges and subsequent pharmacokinetic/pharmacodynamic (PK/PD) issues [2,4]. HGSC is characterized by widespread chromosomal instability and nearly ubiquitous TP53 mutations [4,5]. Recent single-cell genomic studies have shown that whole-genome doubling (WGD) events promote clonal diversification, immune evasion, and therapeutic resistance [4].

First-line treatment for advanced OC typically involves cytoreductive surgery followed by platinum-based chemotherapy combined with taxanes [6]. While the majority of patients achieve initial remission, over 70% relapse due to the emergence of platinum-resistance [6,7]. Recent years have witnessed the incorporation of targeted and biologic agents into therapeutic regimens. *PARP* inhibitors (e.g., olaparib, niraparib, and rucaparib) have become a revolutionary maintenance therapy for Homologous Recombination Deficiency (HRD) positive tumors, whereas antiangiogenic agents such as bevacizumab improve progression-free survival by modulating tumor vascularization [8]. Immunotherapy, though less successful in unselected OC populations, remains a promising avenue when integrated with biomarker-driven selection or combination strategies [7]. Despite significant advances in molecular characterization and targeted therapies, translating preclinical findings into durable clinical benefit remains limited, underscoring the need for predictive experimental systems that recapitulate the PK complexity and molecular diversity of tumors. Achieving therapeutically relevant intratumoral concentrations is notoriously challenging: irregular blood vessel perfusion, elevated interstitial pressure, extracellular matrix barriers, and varying cellular uptake and efflux are factors that hinder uniform drug penetration [7]. 

**Figure 1:**
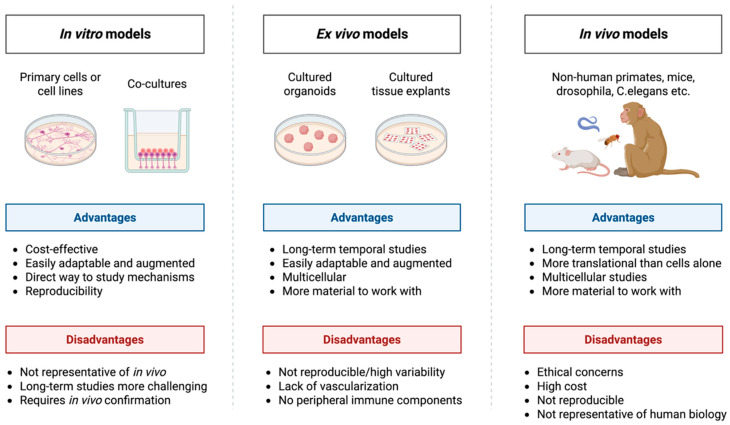
Comparison of *in vitro*, *ex vivo*, and *in vivo* models highlighting advantages, limitations, and translational relevance. Two-dimensional cultures enable high-throughput screening and ease of handling; however, lack the complexity of *in vivo* systems. PDOs better preserve cell–cell and cell–matrix interactions, spatial architecture, and nutrient/oxygen gradients than 2D cultures, resulting in more physiologically relevant drug responses. PDX models provide systemic context and preserve tumor heterogeneity, yet they are time-consuming, costly, and limited by interspecies differences. PDOs frequently outperform 2D cultures in predicting patient responses and serve as a valuable complement to PDX and clinical studies. Integration of organoid-based data with mechanistic pharmacokinetic modeling (e.g., PBPK and IVIVE) and AI/ML approaches enhances translational predictivity and may reduce the need for animal experimentation. AI—artificial intelligence; IVIVE—*In vitro*–*in vivo* extrapolation; ML—machine learning; PBPK—physiologically based pharmacokinetic; PDO—patient-derived organoid; PDX—patient-derived xenograft; 2D—two-dimensional. Created in BioRender. Lima, A. (2026) https://BioRender.com/xdaqkcv.

Despite significant advances in cancer research, a persistent gap remains between preclinical models and clinical outcomes. Conventional two-dimensional (2D) cultures fail to recapitulate tumor architecture and microenvironmental gradients, while *in vivo* models, although more physiologically relevant, present limitations related to cost, scalability, and interspecies differences. To better illustrate these challenges, Figure 1 summarizes the key discrepancies between *in vitro* and *in vivo* systems, highlighting the limited ability of current models to accurately reproduce heterogeneous drug distribution and response. This gap is particularly relevant in the context of solid tumors, where spatial constraints critically influence therapeutic efficacy.

Traditional 2D cell cultures, while valuable for an initial drug screening, fail to recapitulate the three-dimensional (3D) structure, cellular heterogeneity, and dynamic gradients that control drug penetration, metabolism, and resistance *in vivo* [7,8]. Similarly, animal models, while indispensable for studies of drug efficacy against tumors and systemic PK studies, often exhibit interspecies metabolic discrepancies that hinder translational relevance [7,8].

Preclinical systems have been used to apply PK/PD principles under controlled dosing, beginning with animal models that enable the collection of serial blood samples and tissue samples from normal tissues and tumors [9,10]. Specifically in OC, subcutaneous xenografts are widely used, although this model does not exhibit transcoelomic spread nor malignant ascites (MA) accumulation [11]. Orthotopic intraperitoneal models using both cell line–based or patient-derived xenografts (PDXs) better mimic mesothelial adhesion, peritoneal dissemination, and the MA milieu, thus allowing assessment of intraperitoneal delivery, spatial drug distribution, and exposure–response within the metastatic niche [9,10,11,12,13,14]. Humanized mouse models, which reconstitute components of the human immune system, further enable the determination of PK/PD for immunomodulatory agents and reveal bidirectional interactions between drug disposition and the tumor–immune microenvironment (TIME) [15,16]. Thus, these systems—though resource-intensive and variable—provide essential foundations for PK/PD research, which can be combined with emerging human-relevant platforms such as patient-derived organoids (PDOs). In fact, recent OC studies demonstrated how intraperitoneal paclitaxel nanoformulations and targeted nanocarriers have been profiled for pharmacokinetics, biodistribution, and efficacy using PDOs [11,17]. However, because PDOs only partially replicate the MA-conditioned dissemination and human stromal environment, complementary human-relevant platforms are needed.

Over the past decade, PDOs have emerged as a powerful intermediate model, bridging the gap between simple *in vitro* systems and complex *in vivo* tumors [18,19]. This self-organizing 3D culture retains the genetic, histologic, and functional features of the patient’s tumor, offering a powerful platform for studying intra-tumoral drug distribution, metabolic processing, and response dynamics under near-physiological conditions [19].

PDOs are promising models that recapitulate tumor architecture and heterogeneity and have strong applicability to epithelial cancers, including colorectal, pancreatic, and breast cancers [18]. However, their performance varies depending on tumor context, particularly in cancers that rely heavily on stromal, vascular, or immune components, such as pancreatic ductal adenocarcinoma, hepatocellular carcinoma, and immunotherapy-responsive colorectal cancer. PDO-based approaches are particularly informative for solid tumors, where spatial organization and extracellular matrix barriers influence drug exposure and response. In this context, ovarian cancer represents a particularly relevant setting, as it is an epithelial tumor with well-established PDO models that is already known to preserve key tumor features [19,20]. In addition, peritoneal dissemination, malignant ascites, and heterogeneous intratumoral drug penetration are central determinants of treatment response, underscoring the relevance of PDOs for studying resistance mechanisms in this disease.

In OC, PDOs preserve histopathological, genomic, and drug-response fidelity of the original tumors [20]. Their 3D structure enables dose–response assays, temporal sampling, and molecular analysis in a setting that more closely mimics *in vivo* behavior than 2D and 3D cell cultures. Integration with technologies such as microfluidic “organoid-on-a-chip” systems has further advanced human physiological analysis by enabling dynamic perfusion, gradient formation, and controlled media exchange [21]. When combined with multi-omics profiling, high-resolution imaging, and stromal/immune co-cultures, PDO platforms are becoming increasingly sophisticated and relevant for clinical translation [22].

While PDOs have been widely applied for drug screening and molecular profiling, their integration into PK and PD modeling remains underdeveloped. This review explores the advances at the intersection of PK, PD, and PDOs modeling, emphasizing how these systems can capture drug transport, metabolism, and spatial concentration gradients to inform key PK/PD parameters, support *in vitro*–*in vivo* extrapolation (IVIVE), and enable mechanistic models for drug delivery and response prediction. By integrating insights from quantitative pharmacology, tumor biology, and hybrid platforms (e.g., organoid-on-a-chip and computational modeling), this review highlights how PDOs are enhancing translation predictivity and guiding the next generation of precision therapeutics for OC.

## 2. Pharmacokinetics and Pharmacodynamics in Oncology

Pharmacokinetics (PK) describes how a drug is absorbed, distributed, metabolized, and eliminated by the body, whereas pharmacodynamics (PD) refers to the biological effects of a drug at its site of action [23]. Together, PK/PD provide a quantitative framework that links exposure to efficacy and toxicity [24,25]. PK/PD models capture interpatient variability in key exposure parameters (e.g., clearance and volume) and relate exposure metrics (AUC, C_max_, and time-above-threshold) to therapeutic outcomes and adverse-event markers such as neutropenia [26]. Mechanistic PK/PD models represent drug distribution through physiological compartments parameterized by tissue volumes/perfusion/permeability and drug-specific properties [23], and can incorporate tumor-relevant features (e.g., MA, mesothelium, and tumor nodules) to improve dose prediction, support first-in-human studies, assess drug–drug interactions, and inform population-specific adjustments [27,28]. Exposure–response modeling can also accommodate resistance mechanisms (e.g., efflux upregulation, enhanced DNA repair, hypoxia-related tolerance, and stromal shielding) that shift PD curves and reduce maximal effect [24]. Since many anticancer agents have narrow therapeutic windows, even modest changes in exposure can tip the balance between benefit and toxicity [24,29], making quantitative PK/PD essential for regimen optimization. Importantly, these models depend on reliable input parameters from preclinical systems—an area where PDOs can provide high-fidelity and patient-specific data [25,27].

In the context of PDO models, PK and PD concepts must be interpreted at the tumor level. PDOs do not recapitulate systemic PK, such as organ-level distribution or hepatic metabolism. Instead, PDO models enable the study of intratumoral drug penetration, local accumulation, and diffusion within a three-dimensional tumor-like structure [30].

Likewise, PD readouts in PDOs reflect cellular responses to drug exposure, including changes in viability, signaling pathways, and transcriptional states. Although this poses a limitation for modeling systemic metabolism, it offers an additional advantage in dissecting localized drug effects within tumor tissue. Integration with complementary approaches, such as PBPK, IVIVE, or multi-organ systems, is required to capture the full PK/PD landscape [31].

**Figure 2:**
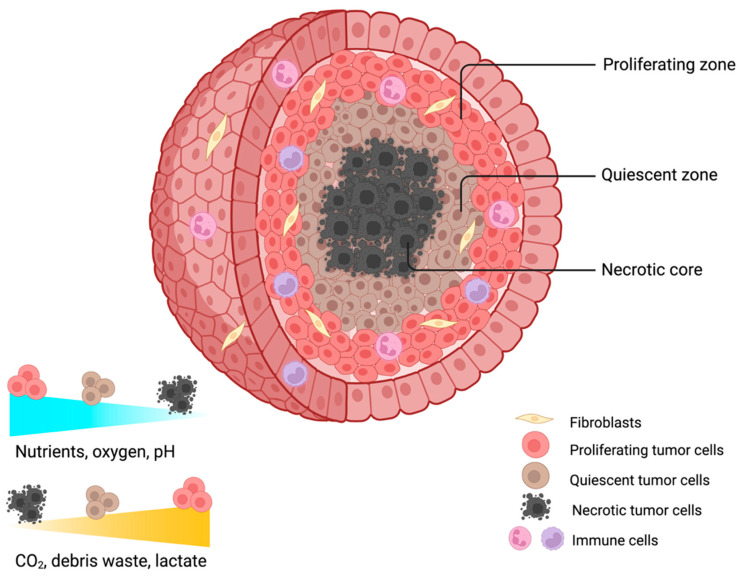
Spatial drug gradients and sampling regions in PDOs. Schematic cross-section of a PDOs illustrating the formation of drug concentration gradients from the periphery to the core. Outer regions receive greater drug exposure, while the central core remains relatively underdosed. The PDOs comprise concentric zones: outer (high drug concentration), intermediate (moderate drug concentration), and core (low drug concentration). Drug diffusion, metabolism, and efflux activity contribute to these gradients. Sampling these zones enables quantification of regional drug and metabolite levels, supporting analyses of PK, resistance mechanisms, and therapeutic efficacy. PDOs—patient-derived organoids; PK—pharmacokinetic; pH—measure of solution acidity/alkalinity. CO_2_—carbon dioxide. Created in BioRender. Lima, A. (2026) https://BioRender.com/zjzobd0.

Solid tumors add a layer of complexity due to spatial/temporal heterogeneity and microenvironmental barriers: gradients of oxygen/nutrients/pH, hypoxic or quiescent zones, and Extracellular matrix (ECM) and vasculature-driven transport limitations can reduce penetration and alter local PK [32,33]. The 3D architecture of PDOs inherently generates intratumoral drug gradients, in which peripheral cells experience higher exposure than central cores; sampling concentric zones (outer/intermediate/core) helps map the distribution and identify underdosed niches (Figure 2) [32,33]. Spatially resolved methods (e.g., imaging mass spectrometry and autoradiography) enable profiling of drug distribution within 3D structures [34,35]. In addition, PDO can express drug-metabolizing enzymes, supporting quantification of intratumoral conversion by comparing metabolite levels in lysate versus medium, while transporter activity (e.g., *P-gp*, *BCRP*, and *MRP*) can be assessed through accumulation assays to clarify how efflux limits intracellular drug levels and contributes to chemoresistance [36,37]. Thus, while advances in 2D culture and animal models have improved understanding of fundamental pharmacological principles, neither can fully capture the interplay between spatial architecture and dynamic PK/PD responses. PDOs, by capturing tumor heterogeneity and microenvironmental cues, may offer a unique opportunity to bridge this gap.

Organoid-based models can be broadly classified into different categories, including PDOs, tumor spheroids, and more complex co-culture systems incorporating stromal or immune components. Each model offers distinct advantages: PDOs preserve tumor architecture, genetic heterogeneity, and cell–cell interactions; spheroids provide simplified systems for studying diffusion gradients and drug penetration; and co-culture models enable investigation of tumor–microenvironment interactions [38,39].

Across multiple solid tumor types, including colorectal and pancreatic cancers, organoid systems have been successfully used to study spatially heterogeneous drug distribution and resistance mechanisms driven by limited drug penetration. These studies have demonstrated that three-dimensional tumor organization can create pharmacological gradients that are not recapitulated in conventional two-dimensional cultures, leading to the survival of resistant cell populations [30,40].

In this context, ovarian cancer represents a particularly relevant setting for the application of PDO models. The disease is characterized by transcoelomic dissemination within the peritoneal cavity, accumulation of malignant ascites, and growth within a complex extracellular matrix-rich environment. These features contribute to heterogeneous drug exposure across tumor sites and are further compounded by the frequent development of platinum resistance. As a result, ovarian cancer exhibits pronounced spatial variability in therapeutic response, making it especially suitable for investigating tumor-level pharmacokinetic and pharmacodynamic mechanisms using PDO-based approaches [38,39].

Together, the parallels with other solid tumors and the unique dissemination pattern of ovarian cancer support the use of organoid models as a physiologically relevant platform to study drug response heterogeneity and resistance mechanisms.

## 3. Ovarian Cancer: Biological and Pharmacological Landscape

**Figure 3:**
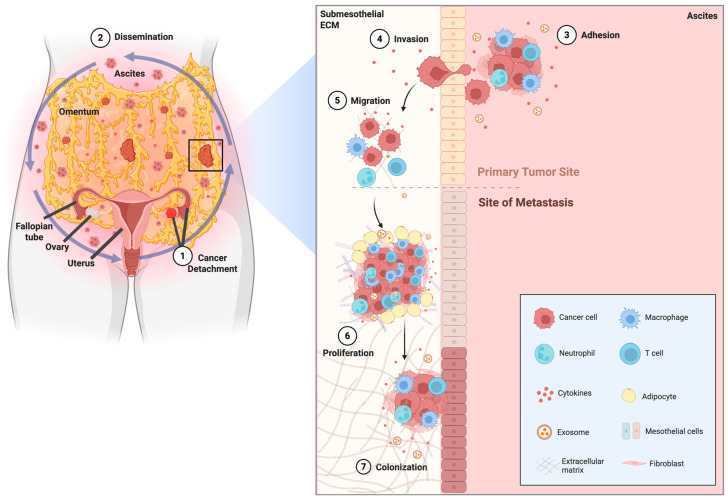
Transcoelomic dissemination driven by malignant ascites in ovarian cancer. Malignant ascites (MA) is a complex tumor-supportive fluid composed of cancer cells, immune cells, mesothelial cells, fibroblasts, adipocytes, soluble factors, and extracellular vesicles, all of which contribute to ovarian cancer progression and metastatic spread within the peritoneal cavity. During transcoelomic dissemination, ovarian cancer cells first detach from the primary tumor site as single cells or multicellular aggregates (1, detachment) and are transported through the peritoneal fluid to distant intraperitoneal sites (2, dissemination). These cells then interact with and attach to the mesothelial surface (3, adhesion), followed by penetration through the mesothelial layer and invasion of the submesothelial extracellular matrix (4, invasion). After successful invasion, tumor cells continue to move through the metastatic niche (5, migration), undergo expansion (6, proliferation), and ultimately establish metastatic implants at secondary sites such as the omentum (7, colonization). MA also provides cytokines, exosomes, and stromal support that facilitate survival, immune modulation, and metastatic outgrowth. ECM—extracellular matrix; MA—malignant ascites; OC—ovarian cancer. Created in BioRender. Lima, A. (2026) https://BioRender.com/f78ejp9.

A critical challenge in OC therapy is that systemic drug exposure often poorly reflects intratumoral concentrations. The peritoneal cavity and MA constitute a permissive, pro-inflammatory niche in which a mesothelial lining over collagenous stroma—with activated fibroblasts, blood vessels, and lymphatic cells—interacts with other cellular and acellular components to shape drug distribution, diffusion, and cellular uptake [41,42]. The cellular fraction includes highly tumorigenic cancer cells, diverse T-cell subsets, tumor-associated macrophages, and other host cells [43,44,45,46]. The acellular fraction is enriched in soluble mediators, bioactive lipids, cytokines/chemokines, extracellular vesicles, prominently stromal cell-derived factors, *IL-6*, *IL-8*, monocyte chemoattractant protein-1, CCL5/CCL7, *TGF-β1*, *TNFα*, and fibroblast growth factor [41,46,47,48]. Collectively, these components present structural and molecular features that generate biophysical and biochemical barriers, restrict drug penetration, modulate cellular responses, and promote chemoresistance (Figure 3) [49,50].

**Figure 4:**
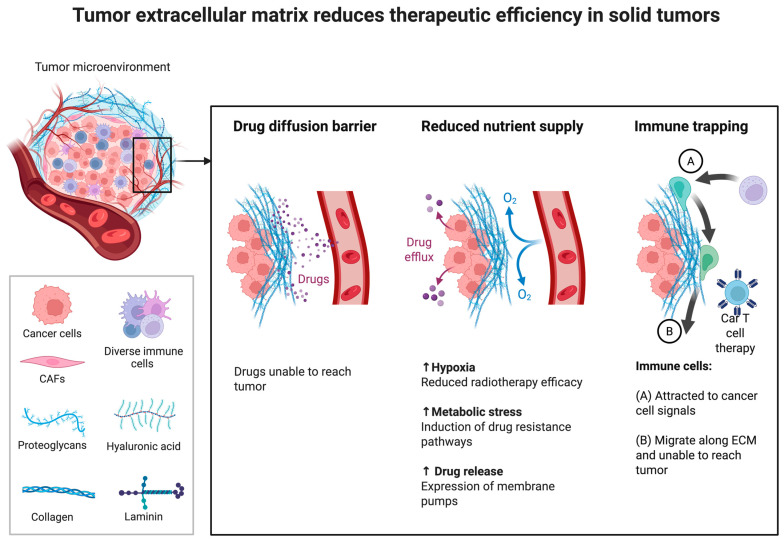
Tumor extracellular matrix (ECM) limits the therapeutic efficacy of anticancer agents in solid tumors. Dense ECM components, including collagen, laminin, hyaluronic acid, and proteoglycans, act as physical barriers to drug penetration (**left**), restrict the diffusion of oxygen and nutrients, thereby promoting hypoxia and metabolic stress (**center**), and hinder immune cell infiltration (**right**). In addition, the ECM may reduce intracellular drug retention, promote drug resistance-associated mechanisms, and impair CAR T-cell and other immune-based therapies by trapping or misdirecting immune cells within the stromal compartment. Together, these features of the tumor microenvironment contribute to therapy resistance and may reduce the predictive value of systemic pharmacokinetic models. Abbreviations: CAFs, cancer-associated fibroblasts; CAR T, chimeric antigen receptor T cells; ECM, extracellular matrix. Created in BioRender. Lima, A. (2026) https://BioRender.com/3zi69gw.

Importantly, the cell-free MA supernatant functions as a metastasis-associated extracellular matrix that modulates viscosity, diffusion, and cellular adhesion, ultimately affecting drug accessibility (Figure 4) [51]. This fluid phase can be leveraged to develop *in vitro* models that better replicate the OC metastatic niche. As a result, the MA TIME not only drives proliferation and immune evasion but also represents a critical determinant of therapeutic response [43].

From a mechanistic standpoint, the cellular and acellular compartments of MA establish a pro-tumorigenic biochemical and biophysical context that enables tumor cell survival in suspension, fosters anoikis resistance, and promotes peritoneal seeding [43]. MA should not be regarded as an epiphenomenon of advanced OC but as a key driver of dissemination and treatment failure. Through transcoelomic spread, tumor cells detach, traverse the peritoneal cavity, adhere to the mesothelium surface, invade submesothelial tissues, and establish secondary implants. This process exacerbates peritoneal carcinomatosis, compromises cytoreductive surgery, and limits chemotherapy efficacy [43]. Therefore, understanding the dual biological and pharmacological roles of MA in OC is essential for designing therapeutic approaches that overcome drug-delivery barriers, disrupt pro-metastatic signaling, and ultimately improve clinical outcomes.

## 4. Limitations of Conventional Preclinical Models

Historically, preclinical research has relied heavily on 2D cell lines and xenograft models. While 2D cell cultures are efficient for high-throughput drug screening, they fail to replicate the 3D cell–cell and cell–matrix interactions essential for realistic PK/PD behavior [22]. Similarly, patient-derived xenografts (PDXs) capture tumor heterogeneity and *in vivo* drug metabolism but are time- and cost-intensive, exhibit murine-specific PK, and often diverge genetically with serial passaging (Figure 1) [21].

Several shortcomings in these preclinical models underscore a key translational gap, as conventional models often fail to accurately predict human drug disposition and therapeutic response. PDO has emerged as a promising alternative, combining the genetic fidelity of PDXs with the scalability and experimental control of *in vitro* systems. Despite extensive plasma PK data available, intratumoral drug distributions remain difficult to quantify. Tumor biopsies are invasive, episodic, and only provide static information; systemic exposure is often used as a surrogate for tumor-level pharmacology—a simplification that may misrepresent local concentrations, particularly when tissue penetration is limited [27]. While animal models allow measurement of tumor and systemic levels, interspecies differences in drug metabolism, drug transporter expression, and diverse aspects of tumor microarchitecture constrain their translational relevance [23].

Human-relevant systems, such as *ex vivo* platforms, offer controlled environments for studying tumor-level kinetics, helping refine PK models and predict clinical outcomes. Integrating systemic PK/PD with tumor-specific and organoid-based data is essential for developing personalized oncology [26].

A comparative understanding of preclinical systems contextualizes the role of PDO within translational PK/PD research. Each model provides distinct advantages depending on the experimental goals—from mechanistic drug studies to whole-body PK assessment (Table 1) [26].

## 5. Patient-Derived Organoids as a Possible Pharmacokinetic Platform

### 5.1. Patient-Derived Organoids

PDO can be generated from primary OC tissues, MA, or metastatic lesions and cultured in ECM scaffolds (e.g., Matrigel^TM^) supplemented with defined growth factors [50,52]. These self-organizing 3D structures preserve key histologic, genomic, and transcriptomic features of the parental tumor, including driver alterations and epigenetic patterns [45,46]. In OC, PDOs reflect patient-to-patient variability and can be expanded long-term with limited genetic drift; establishment rates from surgical specimens can be high (~80% in some cohorts), and MA-PDOs represent disseminated disease biology with platinum sensitivity often aligning with clinical response [52,53].

Functionally, OC PDO supports medium- to high-throughput drug testing across concentration ranges and combinations, including formats that improve exchange and sampling, with viability and mechanistic readouts enabling EC_50_/IC_50_ determination and discrimination between cytostatic and cytotoxic effects [18,54]. Fidelity is typically confirmed by morphology and histopathology (H&E; *p53*, *Ki-67*, and epithelial markers), alongside genomic profiling (targeted/WES; CNV concordance), transcriptomics (scRNA-seq), and epigenetic alignment to the source tumor; importantly, PDO can retain major and minor clonal populations, preserving intratumoral heterogeneity across passages [50,52,55,56,57,58].

### 5.2. Tumor Microenvironment and Drug Transport

A key advantage of PDO is its capacity to reproduce spatial microenvironmental features that shape drug transport, including ECM-driven diffusion barriers and gradients of proliferation and metabolism (e.g., hypoxic cores versus proliferative peripheries). This makes PDO useful for interrogating effective diffusion, binding/retention, and local metabolism, and integration with organoid-on-a-chip platforms adds dynamic perfusion and real-time monitoring under flow, thereby better approximating delivery kinetics. However, standard PDO cultures still lack key TME components (e.g., fibroblasts, immune cells, vasculature, and systemic inputs), and Matrigel-like matrices do not recapitulate native ECM composition, remodeling, or mechanics [45,49,51]. To narrow this gap, co-culture approaches incorporating stromal, endothelial, and immune populations (e.g., T cells, macrophages, and NK cells) are increasingly paired with microfluidic organoid-on-a-chip systems that provide continuous perfusion, shear, and interlinked modules to emulate vascular/lymphatic exchange and multi-tissue coupling [59,60]. Despite these limitations, PDOs offer rapid, scalable, patient-relevant readouts that preserve architecture and heterogeneity, enabling time-resolved sampling of spatial exposure–response. Compared to PDX models, PDOs are faster and require fewer resources, and drug-sensitivity concordance with patients has been reported, supporting their complementary translational value [53]. Combining PDO measurements with physiologically based pharmacokinetic (PBPK)/IVIVE frameworks and selective AI/ML analytics can further improve translational prediction and reduce reliance on animal studies.

### 5.3. Pharmacokinetic Readouts and Modeling in Patient-Derived Organoids

Recent imaging and analytical advances have expanded the set of measurable PK parameters in PDO systems. Fluorescent and radiolabeled tracers allow quantitative mapping of intra-PDO distribution and time-resolved penetration profiles, while live-cell microscopy captures spatiotemporal drug concentration gradients under repeated or pulsed dosing [8]. High-resolution mass spectrometry imaging (e.g., MALDI-MSI) and autoradiographic methods provide label-free or isotopic visualization of administered compounds and metabolites within intact 3D structures, enabling localization of diffusion-limited niches and binding-site barriers [34,35]. In parallel, bulk and micro-region liquid chromatography–mass spectrometry (LC–MS) workflows quantify intra-PDO uptake, metabolic turnover, and retention, thereby enabling inference of diffusion coefficients, intracellular partitioning, and apparent clearance within the 3D matrix [18,54]. For large biologics and antibody–drug conjugates, reaction–diffusion behaviors and saturation of interstitial binding sites can be parameterized directly from spatial profiles to estimate effective permeability and target-mediated distribution [61].

These measurements naturally feed into *in vitro*–*in vivo* extrapolation (IVIVE) pipelines. PDO-derived parameters—diffusion and partition coefficients, apparent uptake/efflux rates, and metabolic kinetics—can be mapped to physiologically based PK/PD tumor compartments to simulate plasma–tumor drug concentration–time profiles and exposure heterogeneity [23,28,62]. Reverse dosimetry then estimates systemic doses required to reproduce PDO-level effective drug concentrations *in vivo*, supporting hypothesis-driven refinement of dose, schedule, and route [63]. When PDOs are embedded in microfluidic organoid-on-a-chip platforms, PBPK-simulated plasma input functions can be used as perfusion waveforms, enabling dynamic exposure experiments that more closely approximate vascular delivery, wash-in/wash-out kinetics, and interstitial convection [56,57,62,63]. Vascularized organoid-on-a-chip systems further extend this approach by introducing perfusable microvessels and controlled shear, which sharpen estimates of flow-limited versus diffusion-limited transport [64].

Integrating organoid PK with PD readouts (e.g., viability, apoptosis, and DNA-damage markers) enables construction of exposure–response models that account for spatial drug gradients, matrix binding, and time delays between drug exposure and effect [25,26]. In practice, PDO-anchored PK/PD modeling offers a mechanistic approach to personalize dosing, assess delivery strategies (including intraperitoneal regimens relevant to OC), and prioritize drug combinations—while providing transparent parameter sets for sensitivity and uncertainty analyses that support translational prediction and, when appropriate, decrease reliance on animal studies [63,65].

### 5.4. Integrative PK/PD Modeling Using Patient-Derived Organoids and Computational and Quantitative Approaches

Physiologically based pharmacokinetic (PBPK) and *in vitro*–*in vivo* extrapolation (IVIVE) models represent complementary approaches to bridge preclinical data and clinical drug behavior. PBPK models integrate physiological parameters with drug-specific properties to simulate systemic distribution, whereas IVIVE approaches translate *in vitro* measurements into *in vivo* predictions [31,66].

In the context of PDOs, these frameworks can incorporate experimentally derived parameters such as drug penetration, cellular uptake, and local response dynamics. However, PDOs primarily inform intratumoral processes rather than whole-body PK. Therefore, PDO-derived data should be integrated into PBPK/IVIVE models as localized inputs, complementing systemic data from other model systems.

The integration of PDO-derived data into computational pharmacology frameworks provides a path from experimental observations to predictive modeling of treatment response. A critical step in this process is demonstrating clinical concordance, ensuring that PDO-derived responses reflect patient outcomes.

A concrete example of its application is reverse dosimetry: an PDO-derived EC_50_ or exposure window (e.g., the free-concentration range that yields DNA-damage signaling) is mapped via PBPK to the systemic dose and schedule required to reproduce equivalent tumor-site concentrations *in vivo* [63]. In OC, PBPK data can compare intraperitoneal and intravenous delivery profiles, and subsequently inform dynamic exposure experiments by perfusing organoid-on-a-chip systems with simulated input functions—reproducing clinically relevant wash-in/wash-out and peak–trough kinetics under flow [56,57,65,67]. Vascularized organoid-on-a-chip platforms extend this further by introducing perfusable microvessels and physiological shear, thereby estimating flow-limited vs. diffusion-limited transport and improving the identifiability of permeability and binding parameters [64].

Spatial drug maps from mass spectrometry imaging or autoradiography in 3D cultures enable fitting reaction–diffusion–binding models to measure effective diffusivity, reversible binding, and sequestration—parameters that are then integrated into PBPK tumor compartments [8,34,35]. For large biologics/ADCs, spatial profiles can be used to estimate target-mediated drug distribution and binding-site barriers, which explain shallow drug penetration and delayed equilibration; these effects can be captured using mechanistic terms for saturable binding and nonlinear clearance [61]. When relevant to OC, PBPK can be configured to evaluate intraperitoneal nanoparticle regimens and compare predicted tumor exposure with organoid-measured uptake/retention, closing the loop between delivery strategy and tissue-level PK [11].

A key translational barrier is assuming that medium concentration equals free drug concentration. PDO—and their matrices—introduce binding and diffusion barriers that lower the pharmacologically available fraction; aligning assay ranges to clinically observed C_max_ and AUC and sampling medium plus organoid lysates over time improves translation by reconstructing drug uptake, steady-state accumulation, and efflux kinetics [8,26,68]. Drug concentration–time metrics (e.g., intracellular AUC, and time-above-threshold) link to PD readouts—viability, apoptosis, *γH2AX*, or transcriptomic changes—to model exposure–response with time delays and spatial effects [25].

Finally, ML augments (but does not replace) mechanistic workflows by extracting phenotypic features from live-cell imaging and learning structure–response mappings from multi-omics measured in the same PDO. These models can impute missing PK/PD parameters (e.g., effective diffusivity conditioned on ECM or transporter expression) and contribute to global sensitivity/uncertainty analyses of PK/PD simulations, accelerating virtual clinical trials that explore dosing, formulation, and combinations in silico [56,57,67,69]. Together, these integrative strategies connect drug exposure to effect in human-relevant tissue contexts and create a transparent pathway to individualized dosing, rational drug combination design, and reduced reliance on animal studies.

### 5.5. Translational Potential of Patient-Derived Organoids for Clinical Outcomes

A critical step in validating PDO-based PK/PD modeling is demonstrating clinical concordance—i.e., that PDO-derived pharmacologic responses mirror patient outcomes. Several studies in OC have shown strong correlations between *ex vivo* drug-sensitivity profiles and *in vivo* therapeutic responses, particularly for platinum drugs, *PARP* inhibitors, and novel targeted agents. Such evidence supports the use of PDO as individualized testbeds for therapeutic optimization. When integrated with PK modeling, PDO can help predict optimal drug exposure windows, identify subpopulations likely to benefit from specific regimens, and flag potential drug toxicity risks through cross-tissue comparisons with healthy organoid models [6,7,63].

Furthermore, dynamic modeling approaches—such as time-dependent PK/PD simulations—allow the capture of adaptive resistance mechanisms within PDO, including the activation of alternative survival pathways or metabolic reprogramming. These insights not only enhance drug development but also provide a framework for adaptive clinical trial design, in which real-time PDO feedback informs therapeutic adjustments [22].

To translate PDO-derived data into predictions of *in vivo* behavior, mechanistic modeling is combined with advanced *in vitro* platforms. PBPK models represent the body as a system of perfused, interconnected compartments, including explicit representations of tumor and interstitial spaces. Parameters measured in PDO—such as diffusion coefficients, metabolic turnover, and transporter-mediated drug uptake/efflux—can be incorporated into these tumor compartments to enhance mechanistic fidelity [21]. IVIVE, particularly via reverse dosimetry, then estimates the systemic dose required to reproduce *in vivo* tissue drug concentrations equivalent to those observed in PDO [63].

When integrated experimentally, organoid-on-a-chip systems can be perfused with PBPK-simulated plasma input functions, creating clinically relevant, time-varying exposures and calibrating model predictions under dynamic flow conditions [56,57,67]. AI/ML can support these efforts by automating image-based phenotyping and aiding parameter inference, serving as a complementary tool rather than a replacement for the mechanistic IVIVE/PBPK workflow [56,57,67].

Scaling from *in vitro* to *in vivo* requires careful physiological adjustment. PDO metrics (e.g., metabolic clearance per protein) must be scaled to whole-organ clearance using established conversion factors, with corrections for plasma protein binding, perfusion, and tissue distribution kinetics [28,62]. A typical pipeline begins with PDO, drug metabolism, and drug efflux data, translates these into equivalent free-drug concentrations, and uses PBPK to estimate systemic dose and to simulate drug concentration-time curves in tumor and normal tissues. These simulations then inform PD models, which predict tumor response and normal tissue toxicity, and facilitate exploration of therapeutic windows [24,25].

Organoid-on-a-chip platforms enhance this method by incorporating dynamic perfusion, gradient generation, and fluid flow, thereby better simulating interstitial transport and more accurately reflecting *in vivo* conditions than static cultures. Additionally, real-time measurement of drug and metabolite levels both inside and outside cells provides crucial data for PK/PD calibration [54]. Networks of interconnected PDO units can model metastatic dissemination or intertumoral spread, supporting multiscale modeling across compartments [70].

Although IVIVE is well-established in toxicology and drug metabolism, its adoption in oncology remains under development. Quantitative IVIVE (QIVIVE) combines high-throughput drug screening, mechanistic modeling, and omics-informed parameters to deliver more personalized predictions [71]. In PDO-based oncology, transcriptomic and proteomic signatures (e.g., transporter/enzyme expression) can refine model parameters [71,72]. Nevertheless, uncertainties remain, including differences between PDO and *in vivo* diffusion, deviations in enzyme/transporter expression, and microenvironmental influences [69]. These features must be addressed using sensitivity analysis, Bayesian estimation, and uncertainty propagation.

Integrating PDO systems with PK/PD modeling offers mechanistic, tumor-focused predictions, reduces invasive sampling, and enables virtual dose exploration. Challenges include parameter variability, incomplete physiology modeling, limited standardization, computational complexity, and the need for validation. Uncertainty in key assumptions indicates where future research is needed.

## 6. Challenges and Limitations

Despite their significant potential, PDO-based models present several limitations that must be critically considered. A major constraint is the absence of systemic physiology, including liver-mediated metabolism and immune system interactions, which are essential components of drug response *in vivo* [66]. Consequently, PDOs cannot fully capture key PK processes such as drug biotransformation and clearance.

In addition, variability in organoid generation, culture conditions, and extracellular matrix composition poses challenges for reproducibility and standardization. Importantly, the lack of vascularization and limited stromal representation further restricts accurate modeling of drug delivery and microenvironmental interactions, with drug transport largely dependent on passive diffusion [30]. Importantly, PDO models also lack a fully functional immune component, which plays a critical role in shaping tumor evolution, therapeutic response, and the emergence of resistance. While some advanced organoid systems incorporate stromal elements or autologous components, immune cell populations are typically absent or not maintained over time, limiting the ability to recapitulate tumor–immune interactions. This represents a significant constraint, particularly when investigating mechanisms of resistance or evaluating therapies influenced by immune-mediated effects.

These limitations directly impact the interpretation of PK and PD data, particularly when extrapolating findings to clinical settings. Moreover, the absence of standardized frameworks and regulatory validation remains a barrier to clinical implementation. Efforts to develop co-culture and immune-enhanced organoid systems are ongoing, but these approaches remain technically challenging and are not yet standardized. Therefore, PDOs should be viewed as a complementary platform that provides high-resolution insights into tumor-specific drug responses, rather than as a standalone predictive system.

### 6.1. Biological and Technical Variability

Significant variability stems from differences in tissue origin, isolation methods, extracellular matrix composition, and culture conditions. Even within a single patient, sampling different tumor regions can produce PDOs with varied morphologies and drug responses. Prolonged culture periods, genetic drift, and selection pressures may impact the model’s accuracy. As a result, PDOs from the same tumor might differ in growth rates, gene expression, transporter and metabolic activity, and pharmacologic response [50,52]. 

The intra- and inter-PDO heterogeneity, along with variability between patients, makes parameter estimation more difficult and limits the generalizability of findings. Inside each PDO, factors such as drug diffusion gradients, nutrient limitations, and diverse cellular states create additional spatial variability that bulk lysate analyses might obscure [32,33].

Advanced modeling and experimental strategies such as global sensitivity analyses, nonlinear mixed-effects modeling, and spatially resolved sampling can partially address these issues, though uncertainty cannot be fully eliminated [69]. Standardization, therefore, remains essential. Harmonized protocols for PDO derivation, media formulation, and quality control are being established to enhance reproducibility across laboratories. In parallel, biobanking initiatives cataloging genomic and pharmacologic metadata will enable systematic cross-study comparisons and meta-analyses.

### 6.2. Modeling Limitations

PDO inherently lacks vasculature and depends entirely on passive diffusion, precluding accurate modeling of convective drug transport and perfusion-dependent delivery [73,74]. As they grow, central hypoxia or necrosis can develop, further restricting drug penetration. PDO also fails to replicate systemic PK processes, including hepatic and renal metabolism, plasma protein binding, systemic clearance, and inter-organ interactions [75,76].

Standard PDO cultures still lack a fully reconstituted TME, and ECM scaffolds cannot reproduce native ECM composition, remodeling, or mechanics. To narrow this gap, researchers co-culture PDOs with stromal and immune populations—including autologous cells isolated from the same patient (e.g., cancer-associated fibroblasts, endothelial cells, tumor-infiltrating lymphocytes, and peripheral blood mononuclear cells)—in conventional 3D matrices [59,60,77]. Then, complementary organoid-on-a-chip platforms add continuous perfusion to emulate vascular/lymphatic flow and inter-tissue coupling [12,59,60,77,78]. Protocol variability (ECM composition, growth factors, passaging, and operator technique) still introduces inter-laboratory inconsistency, and scaling from microscopic PDO to macroscopic tumor masses brings nonlinear diffusion and binding effects that are difficult to extrapolate [78].

Quantifying spatial drug gradients, metabolite flux, transporter kinetics, and localized PD outcomes remains technically challenging; methods such as imaging mass spectrometry or laser microdissection are informative but labor-intensive and low-throughput. Integrating drug concentration–time data with biologic endpoints (e.g., viability, apoptosis, and biomarker induction) adds another layer of complexity, as molecular responses often lag behind drug exposure. This requires delay compartments or other dynamic structures in PK/PD models [25,26].

Nonlinear dose–response relationships, threshold effects, and saturable kinetics further complicate the simple model [8]. Drug-matrix interactions may reduce free drug availability, invalidating assumptions based on nominal drug concentrations [9]. Without corrections, PK/PD models may overestimate or underestimate drug potency and efficacy. Addressing these challenges demands careful experimental design and advanced modeling strategies. Although PDOs reproduce many features of the TME, they still lack key systemic influences on PK, such as vascularization, immune interactions, and whole-body metabolism, limiting their ability to fully capture ADME dynamics. To overcome these constraints, hybrid systems combining PDO with organ-on-a-chip and microfluidic technologies are emerging. These “multi-organ” microphysiological systems enable perfused co-culture of endothelial, immune, and stromal components, enabling dynamic modeling of drug transport, clearance, and immune-mediated effects—bringing *in vitro* pharmacology closer to human physiology [59,60,77].

### 6.3. Regulatory and Translational Considerations

The incorporation of PDO-based assays into regulatory frameworks remains an ongoing challenge. Agencies such as the U.S. Food and Drug Administration (FDA) and the European Medicines Agency (EMA) require demonstration of analytical validity, clinical validity, and clinical utility before such assays can inform decision-making. The U.S. FDA Modernization Act 2.0 encourages human-relevant alternatives to animal testing, but standardized validation pipelines are still required to ensure data reliability and predictive accuracy [79,80].

Currently, no formal regulatory framework governs PDO-based decision algorithms. For regulatory acceptance, PDO assays must be standardized, reproducible, stable across passages, and validated through blinded multicenter studies that demonstrate value over existing preclinical models [81,82,83].

As these benchmarks are established, PDOs are expected to play a key role in next-generation drug evaluation pipelines, particularly for oncology therapeutics with complex PK behaviors—accelerating the transition toward more human-relevant and ethically responsible testing paradigms.

## 7. Translational and Clinical Perspectives

The clinical translation of PDO-based approaches is an area of growing interest in precision oncology. Several studies have demonstrated correlations between *ex vivo* drug sensitivity and patient outcomes, particularly for platinum agents, *PARP* inhibitors, and targeted therapies in ovarian cancer [38,39,84].

However, integrating PDOs into routine clinical practice remains challenging. Key limitations include the time required to establish organoids, technical complexity, cost, and the lack of standardized protocols. In addition, regulatory approval and large-scale prospective validation are required to support clinical implementation. Moreover, PDOs do not capture systemic PK and PD processes, limiting their predictive capacity in clinical practice. Therefore, PDOs’ most realistic application lies within an integrated approach that combines PDO-derived data with genomic, clinical, and pharmacological information to support patient stratification and treatment selection.

Looking forward, PDO platforms offer significant potential not only for preclinical drug screening but also for personalized drug treatment at the dosing level. In an ideal clinical workflow, freshly resected or biopsied tumor tissue and MA could be rapidly converted into PDO, which are then analyzed for drug uptake, efflux, retention, and dose–response effect. These measurements would yield a tumor-specific PK/PD “fingerprint.” When integrated into patient-specific PBPK/PD models, this information could guide personalized dosing regimens and therapeutic optimization. For this approach to be clinically viable, PDO parameters must be reproducible, predictive of *in vivo* drug exposure, and correlate with improved patient outcomes, while being easy to implement [12,85].

Evidence supporting this translational potential is growing. Indeed, a meta-analysis of 17 PDO studies across multiple tumor types demonstrated a consistent association between *ex vivo* sensitivity and clinical response [86]. In gynecologic cancers, emerging studies further suggest that PDO-based drug sensitivity may effectively stratify patients by therapeutic response [12,85,87]. Although many of these studies are retrospective and limited by small cohort size, they collectively support the concept of PDO as predictive biomarkers for clinical decisions.

Beyond static prediction, PDO can also support adaptive clinical trial design. Their quick drug-screening ability allows for real-time, biomarker–guided adjustments during a trial—for example, switching to a more effective therapy if a PDO shows drug resistance. Moreover, the scalability of PDO platforms supports systematic evaluation of drug combinations, dose schedules, and sequencing strategies, which can inform personalized arms within umbrella or basket trials.

## 8. Future Considerations

Emerging research is accelerating the convergence of PDO technology with AI/ML, multi-omics profiling, and personalized PBPK frameworks toward patient-specific “digital twins” that can simulate exposure and efficacy in silico prior to treatment. A key frontier is the development of vascularized and multicellular PDO systems, since diffusion-limited delivery in conventional PDO can systematically misrepresent *in vivo* transport [56,57,67]. Incorporating endothelial networks, perfusable channels, and stromal elements can better recapitulate heterogeneous perfusion, endothelium–tumor interactions, and stromal barriers to penetration, thereby narrowing the gap between administered and intratissue-available drug [42,64,82,83].

A major limitation of current PDO-based drug testing is the insufficient integration of molecular and spatial data to explain heterogeneous drug penetration and response. To address this, we propose a multi-omics framework based on three complementary axes: (i) molecular profiling, (ii) spatial resolution, and (iii) functional drug response.

PDOs should be characterized using integrated transcriptomic, proteomic, and metabolomic approaches, combined with spatial technologies to map intramodel drug distribution. These layers should then be linked to functional readouts such as viability and cell-state changes. Computational integration, including network modeling and machine learning, can identify molecular programs associated with differential drug exposure and response, while longitudinal analyses may reveal dynamic adaptations and resistance mechanisms [87,88,89].

Importantly, coupling drug-distribution data with single-cell and spatial transcriptomics would enable the identification and localization of surviving cell populations, clarifying whether resistance arises from poor drug penetration, intrinsic cellular programs, or both [88]. Overall, this strategy provides a practical roadmap to connect drug penetration dynamics with tumor biology, improving the predictive value of PDO models.

Tumor heterogeneity in PDOs encompasses both cellular diversity and spatial organization, which critically influence drug access and resistance. For instance, cells located in poorly penetrated regions may survive and expand due to sub-therapeutic drug exposure, a phenomenon well described in solid tumors [30,40]. Therefore, integrating single-cell RNA sequencing (scRNA-seq) with spatial transcriptomics enables the identification of cellular subpopulations and their spatial distribution, revealing how tumor cell location influences drug response [88].

Recent studies have demonstrated that combining single-cell data with high-resolution spatial platforms, such as Xenium, can map tumor architecture and define cellular niches, linking transcriptional states to regions with differential drug exposure [88,89]. Applying these approaches to PDOs would enable the identification and localization of resistant clones and the characterization of their molecular features.

Importantly, heterogeneous drug penetration should not be interpreted solely as a physicochemical phenomenon but rather in combination with the transcriptional states of surviving cells, as treatment failure may reflect the persistence of biologically distinct subpopulations. A practical strategy for future PDO-based studies is to combine drug exposure, intramodel drug distribution mapping, and matched single-cell and spatial transcriptomic profiling to identify which cell population persists after drug treatment.

Overall, integrating resolved and single-cell approaches into PDO drug testing frameworks spatially is essential to improve the understanding of resistance mechanisms and enhance the predictive value of these models [87,88,90].

In parallel, AI/ML integration is becoming essential as time-lapse imaging, multi-omics data, and high-throughput dose–response data exceed the capacity of manual analysis. Deep learning can automate feature extraction, detect and correct for experimental variability, and infer PK/PD-relevant parameters from phenotypic cues, while also enabling harmonization across laboratories and privacy-preserving model development when data sharing is constrained [56,57,67,72].

Advances in quantitative modeling are poised to strengthen mechanistic translation. PDO-based frameworks can explicitly represent diffusion, uptake, metabolism, and efflux within 3D constructs and become substantially more informative when parameterized with omics data that capture inter-patient variability in transporters and drug-metabolizing enzymes [78]. Reduced-order approaches can retain the key predictive structure while remaining computationally tractable, lowering the barrier to routine use and facilitating integration with PBPK workflows [88]. As these technologies mature, standardized performance metrics (e.g., penetration- and efflux-related indices) may offer pragmatic, early go/no-go criteria for development and enable more comparable benchmarking across platforms and studies [72].

## 9. Conclusions

PDO serves as a transformative bridge between systemic dosing and intratumoral effects, enabling spatial and temporal analysis of drug penetration, metabolism, efflux, and PD responses. These quantitative data can be integrated into mechanistic models, such as PBPK and IVIVE, to more accurately predict *in vivo* behavior.

Integrating PDO-based PK data with AI/ML analytics enables parameter inference, multi-modal data integration, and dosing simulation—advancing model-informed, patient-tailored therapies. This integrated, model-informed pipeline holds strong potential to reduce reliance on animal experimentation, improve IVIVE, and accelerate the design of adaptive, patient-tailored regimens, aligning with the 3Rs framework (Replacement, Reduction, Refinement) and current regulatory trends supporting human-based new approach methodologies.

To fully realize this potential, PDO systems must more faithfully recapitulate the TME. Embedding PDO in ECM-rich, biomimetic matrices that replicate key biochemical and biophysical features (e.g., collagen and laminin composition, hyaluronan content, proteoglycans, and stiffness) is essential. These should be complemented by stromal and immune co-cultures, as well as vascularization or microfluidic perfusion to enhance the fidelity of drug transport, target engagement, and the assessment of immunomodulatory therapies.

Augmented by ECM-faithful matrices, multicellular TIME components, perfusion technologies, and AI-enabled modeling— PDO are poised to evolve from experimental systems into central assets of translational pharmacology. Standardizing protocols, quantifying uncertainty, and validating model predictions against clinical data will further strengthen their predictive power.

Ultimately, integrating organoid PK into systems pharmacology represents a paradigm shift in translational oncology. By recapitulating the structural, genetic, and metabolic complexity of human tumors, PDO provide a near-physiological platform for studying drug distribution, metabolism, and therapeutic response. In the near future, organoid-guided pharmacokinetics may enable real-time prediction of drug response and resistance, linking laboratory research with clinical decision-making.

The convergence of organoid biology, systems pharmacology, and precision medicine thus heralds a new era in which PDO become indispensable tools for rational drug design, dosing optimization, and the development of safer, more effective therapies for OC.

## Figures and Tables

**Table 1:** Comparative framework of preclinical models in pharmacokinetic and pharmacodynamic research. PDO bridges the translational gap between *in vitro* simplicity and *in vivo* relevance, integrating human genetic fidelity with spatial and metabolic complexity. Future hybrid systems that combine PDO with microfluidics and computational PK modeling are poised to deliver unprecedented insights into drug behavior across biological scales. ECM—extracellular matrix; PDO—patient-derived organoid; PK—pharmacokinetic; 2D—two-dimensional; 3D—three-dimensional.

Model System	Biological Complexity	PK/PD Relevance
2D Cell Cultures	Low	Limited (3D gradients or ECM)
Multicellular Spheroids	Moderate	Moderate (simulate diffusion gradients)
Patient-Derived Organoids (PDO)	High	High (microenvironmental relevance)
Patient-Derived Xenografts (PDXs)	Very high	High (systemic PK achievable)
Organoid-on-a-Chip/Multi-Organ Systems	Very high	Very high (dynamic perfusion and systemic modeling)
Model System	Biological Complexity	PK/PD Relevance

## Data Availability

No new data were created or analyzed in this study. Data sharing is not applicable to this article.

## Abbreviations

The following abbreviations are used in this manuscript:

2D	Two-dimensional
3D	Three-dimensional
ADC	Antibody–drug conjugate
ADME	Absorption, distribution, metabolism, and excretion
AI	Artificial intelligence
AI/ML	Artificial intelligence/machine learning
AUC	Area under the concentration–time curve
CAF(s)	Cancer-associated fibroblast(s)
CAR T	Chimeric antigen receptor T cell
CCL5/CCL7	C-C motif chemokine ligands 5/7
CNV	Copy-number variation
C_max_	Maximum (peak) plasma concentration
CO_2_	Carbon dioxide
EC_50_	Half-maximal effective concentration
IC_50_	Half-maximal inhibitory concentration
ECM	Extracellular matrix
EMA	European Medicines Agency
FDA	U.S. Food and Drug Administration
H&E	Hematoxylin and eosin
HGSC	High-grade serous carcinoma
HRD	Homologous Recombination Deficiency
*IL-6*/*IL-8*	Interleukin-6/-8
IVIVE	*In vitro–in vivo* extrapolation
*Ki-67*	*Ki-67* proliferation marker
LC–MS	Liquid chromatography–mass spectrometry
MA	Malignant ascites
ML	Machine learning
NK cells	Natural killer cells
OC	Ovarian cancer
O_2_	Oxygen
*PARP*	Poly(ADP-ribose) polymerase
PBPK	Physiologically based pharmacokinetic (modeling)
PD	Pharmacodynamics
PDO(s)	Patient-derived organoid(s)
PDX	Patient-derived xenograft
PK	Pharmacokinetics
PK/PD	Pharmacokinetics/pharmacodynamics
*p53*	Tumor suppressor protein *p53*
pH	Measure of solution acidity/alkalinity
QIVIVE	Quantitative *in vitro–in vivo* extrapolation
RNA-seq	RNA sequencing
scRNA-seq	Single-cell RNA sequencing
T cell	T lymphocytes
*TGF-β1*	Transforming growth factor-beta 1
TIME	Tumor immune microenvironment
TME	Tumor microenvironment
*TNFα*	Tumor necrosis factor-alpha
TP53	Tumor protein p53
WES	Whole-exome sequencing
WGD	Whole-genome doubling
*γH2AX*	Phosphorylated H2AX (DNA damage marker)
